# Typhoon disaster emergency forecasting method based on big data

**DOI:** 10.1371/journal.pone.0299530

**Published:** 2024-04-25

**Authors:** Hong Huo, Yuqiu Chen, Shiying Wang

**Affiliations:** 1 School of Management, Harbin University of Commerce, Harbin, China; 2 Computer Science and Information Engineering School, Heihe University, Heihe, China; Marshall University, UNITED STATES

## Abstract

Typhoons are natural disasters characterized by their high frequency of occurrence and significant impact, often leading to secondary disasters. In this study, we propose a prediction model for the trend of typhoon disasters. Utilizing neural networks, we calculate the forgetting gate, update gate, and output gate to forecast typhoon intensity, position, and disaster trends. By employing the concept of big data, we collected typhoon data using Python technology and verified the model’s performance. Overall, the model exhibited a good fit, particularly for strong tropical storms. However, improvements are needed to enhance the forecasting accuracy for tropical depressions, typhoons, and strong typhoons. The model demonstrated a small average error in predicting the latitude and longitude of the typhoon’s center position, and the predicted path closely aligned with the actual trajectory.

## 1. Introduction

Typhoons are strong cyclonic vortices (tropical cyclones) that occur on the tropical ocean with a warm central structure. They are vortices of air that move forward and rotate at high speeds around their center in the atmosphere, rotating counterclockwise in the Northern Hemisphere and clockwise in the Southern Hemisphere. Typhoons are strong cyclonic eddies (tropical cyclones) that occur on warm tropical oceans with a central structure. They are forward moving air vortices that rotate at high speed around their center in the atmosphere, counterclockwise in the northern hemisphere, and clockwise in the southern hemisphere. The destructive nature of typhoon disasters has attracted the attention of many scholars. Regarding secondary and derivative disasters, Yin et al. (2022) [1] studied the general characteristics of rainstorm in Fujian caused by typhoons passing through Taiwan Island based on typhoon track and intensity data from 1961 to 2020 and typhoon precipitation data in China. Liu et al. (2023) [2] studied the impact of typhoon attacks on the accuracy of analyst predictions against the background of China. The results indicate that analysts’ exposure to typhoon attacks has led to a decrease in the quality of their predictions.

Many scholars are very interested in typhoon prediction. Gong et al. (2022) [3] established a GA typhoon model based on genetic algorithm (GA) and Jelesnianski typhoon wind model. Observations and analysis were conducted on four typhoon processes and four buoys along the southeastern coast of China. Gong et al. (2022) [4] developed a hybrid Multilayer perceptron (HMLP) neural network and a hybrid genetic expression programming (HGEP) model with switching layers to predict typhoon waves. The results indicate that the number of training typhoons and the similarity between the training typhoon path and the target typhoon have an impact on the prediction results, and the prediction performance is related to the intensity of the typhoon’s impact on the experimental site. Guo et al. (2022) [5] built a risk assessment model based on the disaster risk theory, according to the Areal feature of Guangdong Province and the indicator system selected in previous work. It uses Simulated annealing algorithm and least square method to optimize the model. The optimization model was used to evaluate the harmfulness, vulnerability, disaster resistance, and risk capacity of cities in Guangdong Province from 2001 to 2014.

In addition, some scholars are concerned about the ability to cope with typhoons. Peng et al. (2023) [6] developed a conceptual model to understand the resilience of communities to typhoon disasters from six dimensions: built infrastructure, response effectiveness, self-organization, disaster impact, long-term efforts, and community organization. The results indicate that the PCR of typhoon disasters in Taizhou City is generally good, with differences in different regions. The perception level of disaster impact is the highest, with built infrastructure ranking second, self-organization ranking third, long-term effort ranking fourth, response effectiveness ranking fifth, and community organization ranking lowest. Yu et al. (2022) [7] introduced the concept of impact based forecasting, introduced the implementation and progress of typhoon impact forecasting in TC member countries in recent years, and preliminarily explored measures and directions for strengthening impact based forecasting and early warning services in the future. Hou et al. (2023) [8] considered that the reliability of distribution network power supply is vulnerable to extreme weather events such as typhoons, and proposed a decision-making framework for distribution network resilience enhancement, which is expressed as a two-stage random mixed integer Linear programming (SMILP) model. The first stage of coordinated investment aims to minimize the investment cost of the elasticity enhancement strategy. In the second stage, it is necessary to ensure that the expected recovery operation cost of the comprehensive strategy for all typical scenarios is minimized. Chen et al. (2022) [9] considered the significant losses and impacts caused by extreme meteorological disasters on the distribution network, and believed that for these low probability and high loss events, it is necessary to establish an elastic distribution network to prevent extreme disasters and quickly recover critical loads.

Some scholars have also paid attention to the impact of typhoons on offshore wind turbines. Wang et al. (2022) [10] proposed a multi-stage analysis framework for the impact of typhoons on offshore wind turbines based on the spatiotemporal changes of typhoon wind speed fields. In this framework, an enhanced wind speed field model was used to consider the effects of different typhoon impact stages on the average wind speed profile, typhoon intensity, and spectral characteristics. Ren et al. (2022) [11] used the radial integration method of wind disaster assessment to investigate the wind disasters caused by typhoons landing in China from 2004 to 2020. The typhoon parameters are mainly from the China Meteorological Administration. Firstly, he analyzed two factors that affect typhoon disasters, namely typhoon intensity and spatial scale. After landfall, the intensity and overall spatial scale of the typhoon weakened and decreased, respectively. Secondly, the applicability of the radial integration typhoon disaster assessment method in actual typhoon scenarios was verified using existing typhoon disaster measurement data. In addition, the radial integration method was used to study the changes in wind disasters before and after typhoon landfall in different regions of China. Li Junyu et al. (2023) [12] proposed the algorithm of monitoring typhoon movement which mainly focuses on PWV, and it is very difficult to describe typhoon movement in detail, resulting in insufficient precision. Therefore, based on PWV and meteorological data, an improved typhoon monitoring model is proposed. Ji-Myong Kim [13] used deep learning algorithms to evaluate building losses in typhoon disasters.

Some scholars focus their research on the selection of evaluation indicators. Hideki Tsuji [14] discussed the influence of temperature, water quality and strong typhoon events on climate change. Liang Yutao et al (2023) [15] propose an assessment indicator consisting of prevention and control, emergency response and rapid recovery to handle extreme typhoon events. For typhoon-prone areas, economic indicators are also an important factor [16]. Jaesoo Lim et al (2023) [17] take the change of typhoon frequency and track as an important index.

The above researches have achieved rich results and promoted the research progress of typhoon prediction methods to a certain extent, but most of the researches are to predict the possibility of future typhoon occurrence through data [7]. On the basis of the above research, this paper changes "forecast" to "current measurement", that is, to judge the future trend of the ongoing typhoon, so as to help relevant departments to adopt corresponding emergency strategies in time, so as to control the typhoon to the maximum extent and reduce the damage to people and property caused by the typhoon. Based on the temporal and spatial characteristics of typhoon disasters, this paper optimizes the traditional numerical statistical forecasting model by using big data technology. First of all, Python was used to climb typhoon disaster data and collect typhoon data affecting China in the last ten years from 2009 to 2019, including serial number, longitude and latitude of typhoon center position every six hours, wind speed, direction of movement, central pressure and other information, which was summarized, summarized and sorted out. Secondly, the cyclic neural network model of typhoon disaster prediction is constructed, and the error analysis of each forecasting factor is carried out according to the evaluation index of the model. Finally, through the use case analysis, the typhoon intensity, typhoon position and typhoon trend are analyzed, and the fitting is good and consistent with the reality.

## 2. Methodology

### 2.1 Data crawl

**Fig 1:**
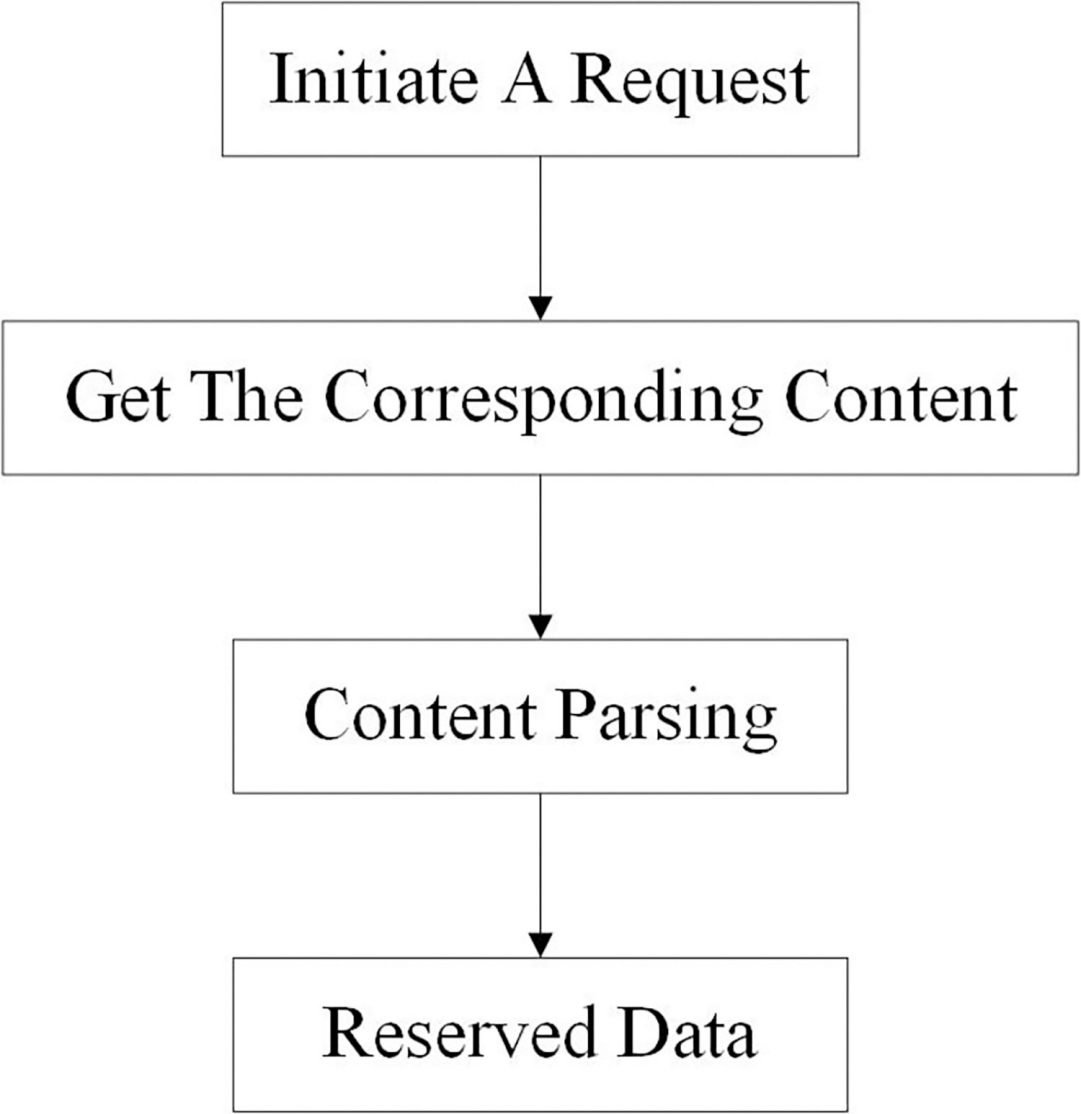
Data crawling process.

The data in this paper are mainly from the Typhoon network of the National Meteorological Observatory. The typhoon system used in this website is issued by the National Meteorological Observatory and can provide the latest real-time typhoon information in time. At the same time, the website combines satellite cloud images, weather radar, rainfall and other contents. The observation data of the last ten years from 2009 to 2019 from the National Meteorological Observation-Typhoon network is selected as the historical typhoon data set in this paper. The data content includes serial number, typhoon name, source, longitude and latitude of typhoon center position every six hours, wind speed, movement direction and typhoon intensity, totaling 13,495 pieces of data. Data crawling adopts Python technology, and the specific process is shown in Fig 1.

Users send their own information to the server through the browser. The server receives the request, analyzes the information and sends the data back to the browser. After receiving the response, the browser analyzes the content and displays it to the user. A crawler simulates a browser sending a request, receiving a response, and extracting useful data. The request is initiated by using http library to send a request to the target site. The information includes pictures, videos, etc. If no error is reported, you can see the basic information of the web page. If the server can respond normally after the crawler sends the request, it will return some information. Data analysis, the web source data analysis, access to their own data information. Finally, the parsing results are stored in an Excel table.

### 2.2 Introduction to data sets

#### 2.2.1 Path data set

Time series is a common data type, and typhoon data set is one kind of time series. The data is collected in sequential order, so correlation is a key feature of time series. By analyzing the time series X(t, t = 0, ±1, ±2…) The study of historical development information can find its past dynamic change rule and then predict its development trend. Time series can be divided into unary time series and multivariate time series according to the number of observed features at the same time. Time series are everywhere, such as the rainfall in downtown Harbin over the past decade, and the country’s gross domestic product data from 2009 to 2019. An example of typhoon track data is shown in Table 1.

**Table 1:** Typhoon track data examples.

Name	Time	Wind Speed	Strength	Central Pressure	Latitude and longitude of the center
0901 Kujira	2009-05-02 02:00:00	13m/s	tropical depression	1004 hectopascal	12.9N/124.2E
0901Kujira	2009-05-02 08:00:00	13m/s	tropical depression	1004 hectopascal	13N/124.3E
0901Kujira	2009-05-02 14:00:00	15m/s	tropical depression	1002 hectopascal	13.2N/124.4E
0901Kujira	2009-05-02 20:00:00	15m/s	tropical depression	1002 hectopascal	13.4N/124.5E

#### 2.2.2 Login data set

This paper analyzes the typhoons that landed in our country from 1949 to 2015 and a study on their characteristics is of great significance for typhoon emergency management of our country. The landfall data of Chinese typhoons are collected from the official website of Shanghai Wind Chasing Team, including 716 pieces of data including Chinese name, English name, landing location, landing time, peak intensity and landing intensity. An example of typhoon landing data is shown in Table 2.

**Table 2:** Examples of typhoon landing data.

English name	Landing location	Landfall time	Peak intensity	Landfall intensity
Sinlaku	Wujie Township, Yilan County, Taiwan Province	2008.9.14	16 degree, 55m/s, 935hPa	14degree, 45m/s, 950hPa
Hagupit	Chencun Town, Dianbai County, Maoming City, Guangdong Province	2008.9.24	15 degree, 50m/s, 940hPa	14 degree, 45m/s, 950hPa
Jangmi	Nanao Township, Yilan County, Taiwan Province	2008.9.28	17+degree, 65m/s, 910hPa	16 degree, 52m/s, 940hPa
Higos	Longlou Town, Wenchang City, Hainan Province	2008.10.3	8 degree, 18m/s, 998hPa	8 degree, 18m/s, 998hPa

#### 2.2.3 Data cleaning

**Fig 2:**
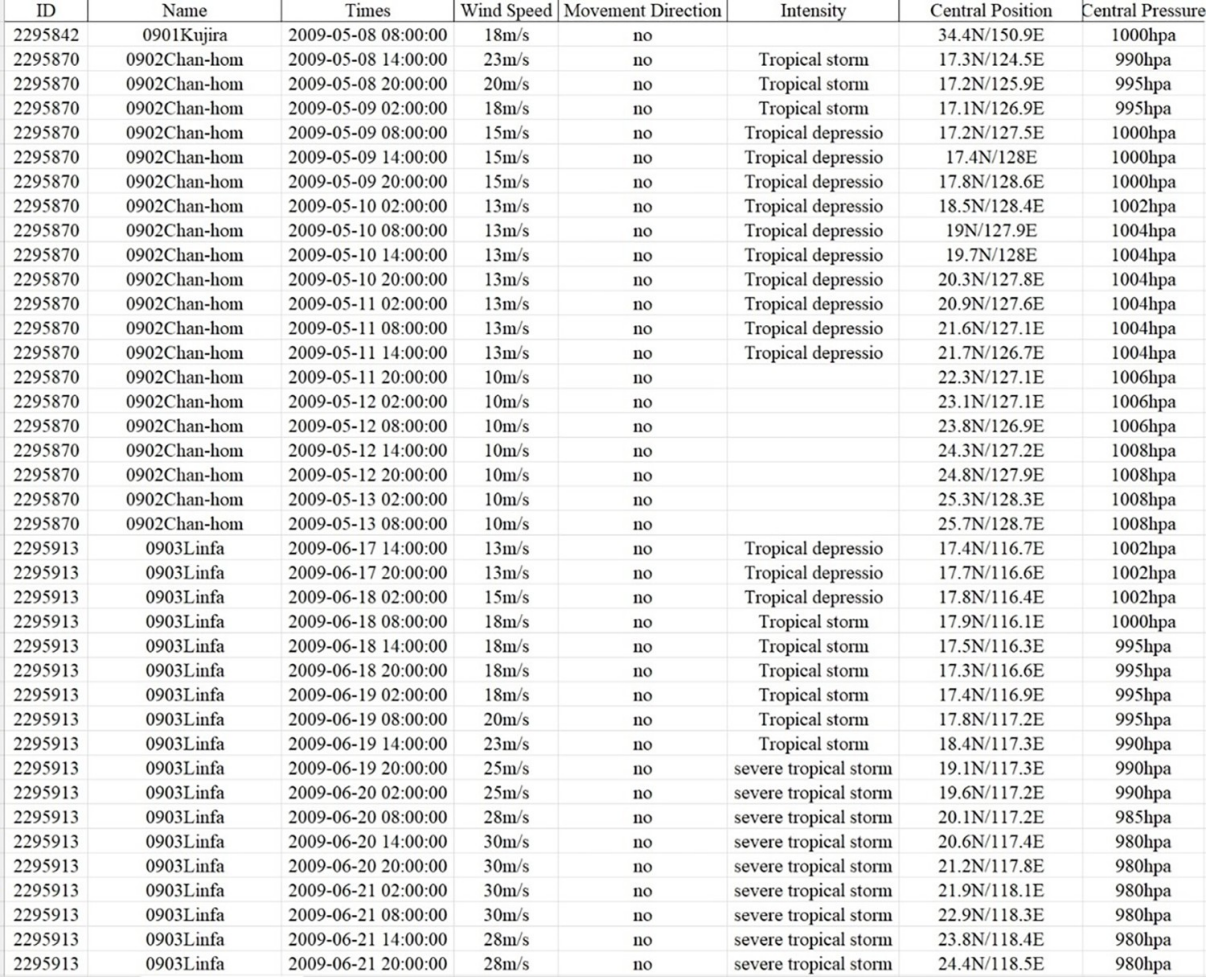
Typhoon data set presentation.

In the process of data crawler acquisition, errors are a normal phenomenon. Therefore, after obtaining the data, it is necessary to clean the data before guiding the model so that the model can obtain more accurate information. Data cleaning is the first step in the whole data analysis process, and also the most time-consuming step in the whole data analysis project. The crawler parses the content and saves the data, which is stored locally in the form of a file. A partial dataset presentation is shown in Fig 2.

As can be seen from the figure above, there are many problems in the data obtained by the crawler, such as missing data, unsuitable format content for importing the model, strength of 64–70 lines, wind direction, etc. Data is cleaned through Excel tables. The process of data cleaning determines the accuracy of data analysis and is the only way to improve the quality of data. This step makes the results of data analysis more reliable.

1. Missing value processing
  Due to various reasons, many data sets in reality contain missing data, which cannot be directly used for training, for which missing values need to be processed. First, the range of missing values is determined, and unnecessary fields are removed, such as the direction and intensity of typhoon track data set.
2. Format processing
  Causes of format and content problems: data is collected manually or filled in by users, content or format generated by different versions of programs is inconsistent, content and format definition of data collected by different data sources is inconsistent, etc. Start with cell split: select menu, data in the separate.
  The format content processing in this paper mainly removes units that should not exist in the content, such as wind speed: m/s, central air pressure: hpa, longitude of central position: E, latitude of central position: N, etc. Press Ctrl+f to get the replacement screen.
3. Logical content processing
  This part of the work is to remove some of the data that can be directly found by simple logical reasoning, to prevent the analysis results from being skewed. Outlier is a kind of special situation that we often encounter in data analysis. The so-called outlier is abnormal data. Sometimes abnormal data is useful to us, and sometimes abnormal data is not only useless to us, but will affect our normal analysis results. After sorting by time, remove id (typhoon number) and time.

#### 2.2.4 Data preprocessing

Data preprocessing is an operation to convert the original data into the format we mine. Before establishing the model, it is usually necessary to conduct standardized processing, and the data falls into a smaller interval to eliminate the influence of orders of magnitude between different dimensions. The common preprocessing method is normalization. Its obvious advantage is that it can improve the algorithm iteration speed and make the objective function converge faster in the model training stage. In this paper, we choose the common normalization method: maximum and minimum standardization to process the data.

Maximum and minimum standardization is also known as deviation standardization, and the formula is shown in Eq (1).

$$X^{*}=\frac{X-X_{\max}}{X_{\max}-X_{\min}}$$ (1)

where X* represents data after normalization, *X* represents raw data, *X*_*max*_, *X*_*min*_ represents the maximum and minimum values in the original data, respectively.

After this step is completed, the minimum data will become 0 and the maximum data will become 1. The data normalized after deviation is closely related to the range of the original data. If the new data changes the maximum or minimum value of the original data, new standardization is needed. After the normalization is completed, the calculation method of attribute similarity is introduced.

## 3. Developing typhoon prediction model based on neural network

### 3.1 Selection of predictive factors

Although the meteorological department has greatly improved the forecast ability of typhoon weather, there are still differences in the forecast ability of different regions. Therefore, studying the impact of typhoon disasters is an important part of typhoon forecast.

Disaster-causing factors are the driving force of disaster losses. They will cause problems such as wind, rainfall and storm surge, and are accompanied by secondary disasters such as floods and landslides, which will cause economic losses and casualties. In this paper, the maximum wind speed was selected as the prediction benchmark through data statistics and combined with the typhoon data characteristics obtained. The longitude and latitude of the central position were used to determine the typhoon position, and the central pressure was used as a variable to measure the change speed of typhoon grade.

### 3.2 Model overview

In this paper, the cyclic neural network prediction model is used, and the output results of ordinary neural network and LSTM are compared to select a better model to explore the method of typhoon data prediction. RNN is a kind of neural network with short-term memory ability [18], which solves the problem that the traditional neural network cannot keep the thinking continuous [19], and the analysis of the development trend of typhoon disaster is a problem of time cycle, and the selection of RNN is conducive to describing the dynamic change process of typhoon disaster. LSTM is a special kind of RNN [20], which is widely used because of its excellent ability to solve the problem that RNN cannot handle long distance dependencies [21], and the solution is to add another state to hold the long-term state. Therefore, this paper chooses LSTM to build a typhoon disaster prediction model [22].

**Fig 3:**
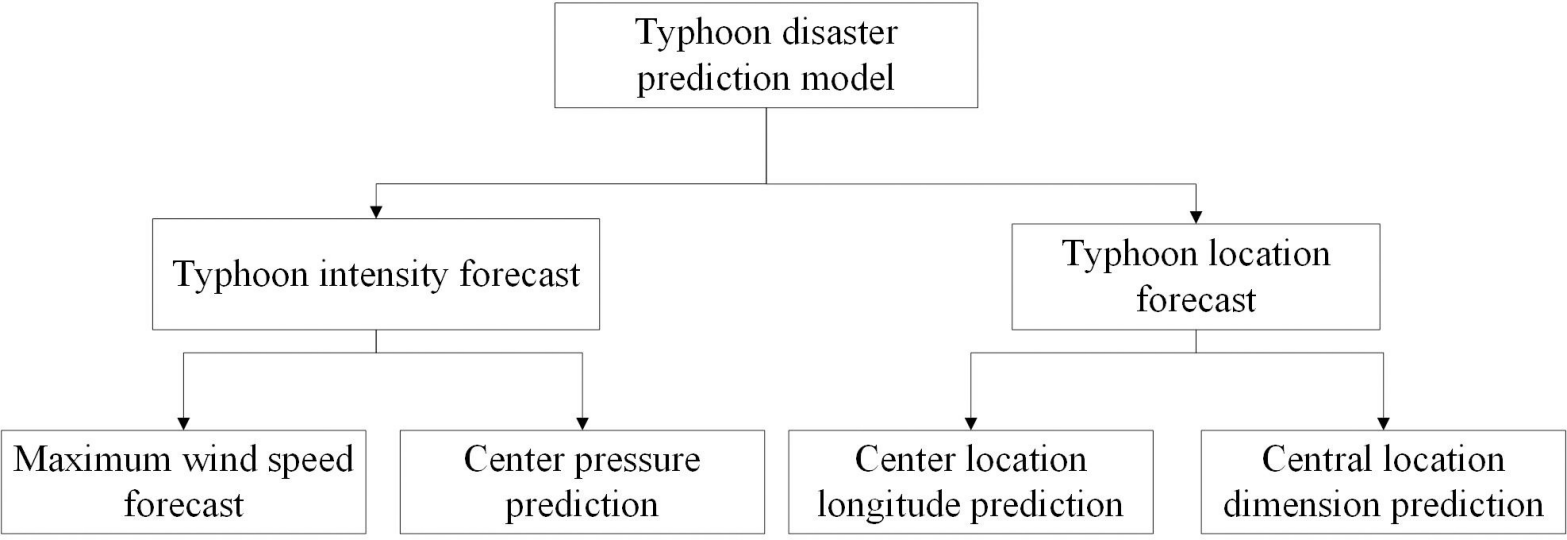
Typhoon disaster prediction model framework.

The framework of typhoon disaster prediction model is shown in Fig 3.

Whether the model can effectively predict the new information of typhoon, the trained model is used to predict the future wind speed, central pressure, longitude and latitude of the central position of typhoon, and the predicted results are compared with the real results. The evaluation indexes of the model chosen here are mean absolute error (MAE) and root mean square error (RMSE).

The average absolute error is the average of the absolute error, which can well reflect the actual situation of the predicted error, so as to find out the difference between the predicted value and the real value. The mean absolute error (MAE) formula [23] is shown in Eq (2).

$$MAE=\frac{1}{n}\sum_{i=1}^{n}\left|f_{i}-y_{i}\right|$$ (2)

where *f* represents the predicted value, *y* that’s the true value.

The root mean square error is the square root of the mean square error. The root mean square error reflects the relationship between the data sample and the real value. The smaller the root mean square error is, the more accurate the prediction of the model is. The formula is shown in Eq (3).


$$RMSE=\sqrt{\frac{1}{n}\sum_{i=1}^{n}{\left(f_{i}‐y_{i}\right)}^{2}}$$ (3)

### 3.3 Model developing

**Fig 4:**
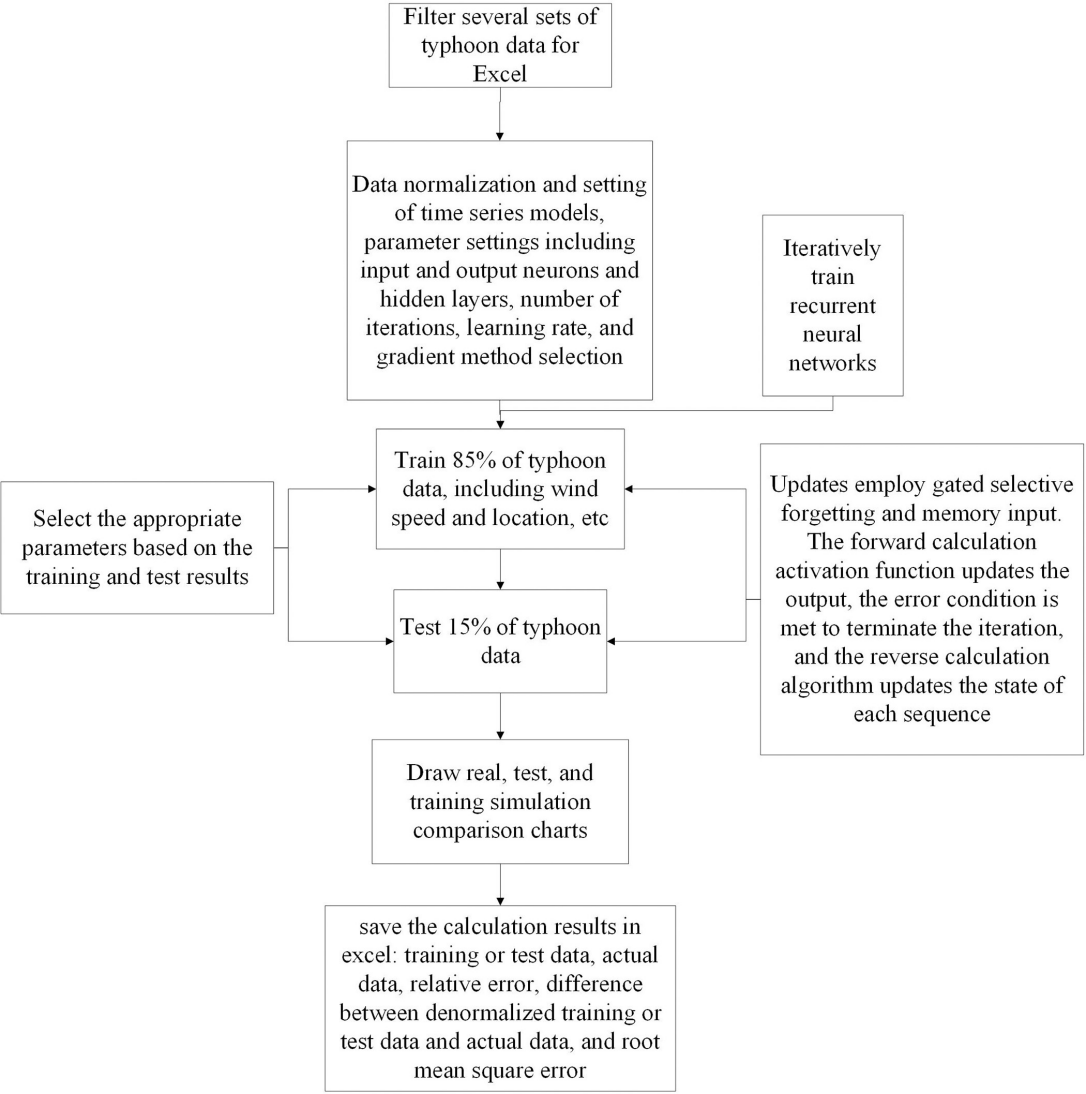
Flow chart of typhoon prediction model.

First, several sets of typhoon data are screened for training purposes. After data preprocessing of the original data set, the data set is divided into training set and test set to make it suitable for the training of the cyclic network. The training set data is input into the cyclic neural network, and the root mean square error (RSME) is used as the loss function for training. The test set is input into the trained model to get the result data. Finally, model verification and performance evaluation, prediction model is used to predict, the predicted value and the real value are compared, and the results are analyzed. The default proportion of the neural network training set is 0.8, that is, 80% is used to train the neural network, and the remaining 20% is used for model validation [24]. Due to the large amount of data in this paper, which is 13,495 articles, the amount of data used for model verification is too large. Therefore, some parameter values need to be specially set to appropriately increase the proportion of training set to 85% and reduce the proportion of verification set to 15%. The flow chart of typhoon prediction model constructed in this paper is shown in Fig 4.

**Fig 5:**
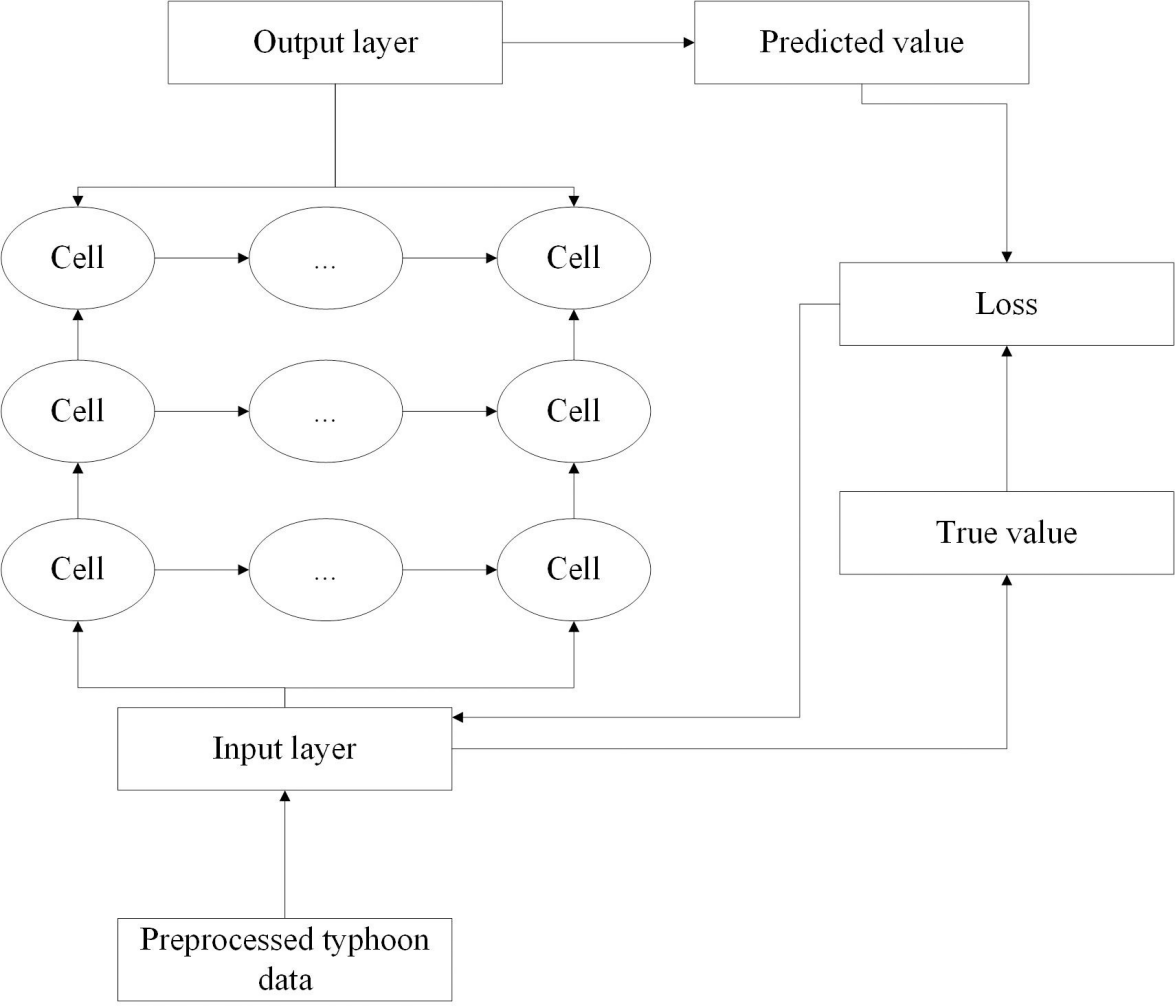
Cyclic neural network structure.

The structure of the cyclic neural network is designed as a three-layer cyclic neural network, namely the input layer, the intermediate layer, and the output layer. The intermediate layer expands from the bottom to the top along the time dimension, and the interconnections between the intermediate layers ensure the transmission of information along the time dimension. The neuron structure of the intermediate layer is different from that of LSTM, and the structure of the cyclic neural network is shown in Fig 5.

Hidden layer forward calculation formula:

The internal calculation formula of BP neural network is shown in Eq (4).

$$h=f(\sum_{i=1}^{n}q_{i}x_{i}‐b)$$ (4)

where *x* represents neuron input, *q* represents weight, *b* represents neuron threshold, *h* represents output.

LSTM neural network completes the internal processing of one neuron through three control gate mechanisms, so that it can form the memory of long-term data in the past.

Oblivion gate: If a cell in the oblivion gate approaches 0, LSTM forgets the stored value of the corresponding unit of the previous cell state. If a cell in the forget gate has a value close to 1, LSTM will remember most of the corresponding values. The sigmoid function of the forgetting gate determines what information to discard from the cell state. Γ is between 0 and 1, and the formula is shown in Eq (5).

$$\Gamma_{f}=\sigma (w_{f}[a^{<t-1>},x^{<t>}]+b_{f})$$ (5)

where *a* represents output at time (*t*-1), the input of this layer at time *x*^<*t*>^, *W* represents weight of each variable, *b* represents learning rate, *σ* represents sigmoid function. The formula is shown in Eq (6).


$$\delta (x)={\left(1+e^{‐x}\right)}^{‐1}$$ (6)

Update gate: Used to determine what new information is stored in the cell state, calculated in three steps: The first step is the calculation result of sigmoid function Γ_f_ of update gate to determine which values we shall update. The second step creates a new candidate value vector based on the tanh function and adds it to the cell state. In the third step, multiply the old cell state by the amnesia gate Γ_f_ to forget part of the old information, and then add Γ_f_ to multiply the new candidate value vector to renew the cell state. The renewal gate formulas are shown in Eqs (7), (8) and (9).

$$\Gamma_{u}=\delta (w_{u}[a^{<t-1>},x^{<t>}]+b_{u})$$ (7)

$${\tilde{c}}^{<t>}=\tanh(w_{c}[a^{<t-1>},x^{<t>}]+b_{c})$$ (8)

$$c^{<t>}=\Gamma_{u}*{\tilde{c}}^{<t>}+\Gamma_{f}*c^{<t-1>}$$ (9)

where, Γ_u_ is between 0 ~ 1, *tanh* is the hyperbolic tangent excitation function, output is the value between -1 and 1.

Where, cell state value when—(t-1);

—Extract the information to be recorded from the input information at time t;

—Updated cell status value.

Output gate: *c* is treated with sigmoid function, Γ_o_ and c are multiplied to obtain the output value at time *t*. The output gate formula is shown in Eqs (10) and (11).


$$\Gamma_{o}=\delta (w_{0}[a^{<t-1>},x^{<t>}]+b_{o})$$ (10)

$$a^{<t>}=\Gamma_{o}*c^{<t>}$$ (11)

Time—based back propagation algorithm (BPTT) is the most common way to train artificial neural networks. The steps are as follows:

First, typhoon wind speed, central pressure and latitude and longitude of central position are input into the input layer, then through the hidden layer, and finally reach the output layer and output the results. This is a forward propagation process.

Back propagation first calculates the error value between the estimated value of the neuron and the actual value, and propagates this error back from the output layer to the intermediate layer, and then to the input layer. Then the actual value and the predicted value are compared through the neural network, and the loss is calculated through the sigmoid function, which is used to optimize the parameters of the neural network to reduce the loss. Finally, the gradient of each weight parameter is calculated, and the weight is updated by gradient descent until convergence.

The modeling is based on the data of Pt+1, Pt+2, Pt+3 to predict Pt+4, including the prediction of wind speed, latitude of central position, longitude of central position and central pressure. This article uses 85% data for training and 15% data for testing.

### 3.4 Modeling results

**Fig 6:**
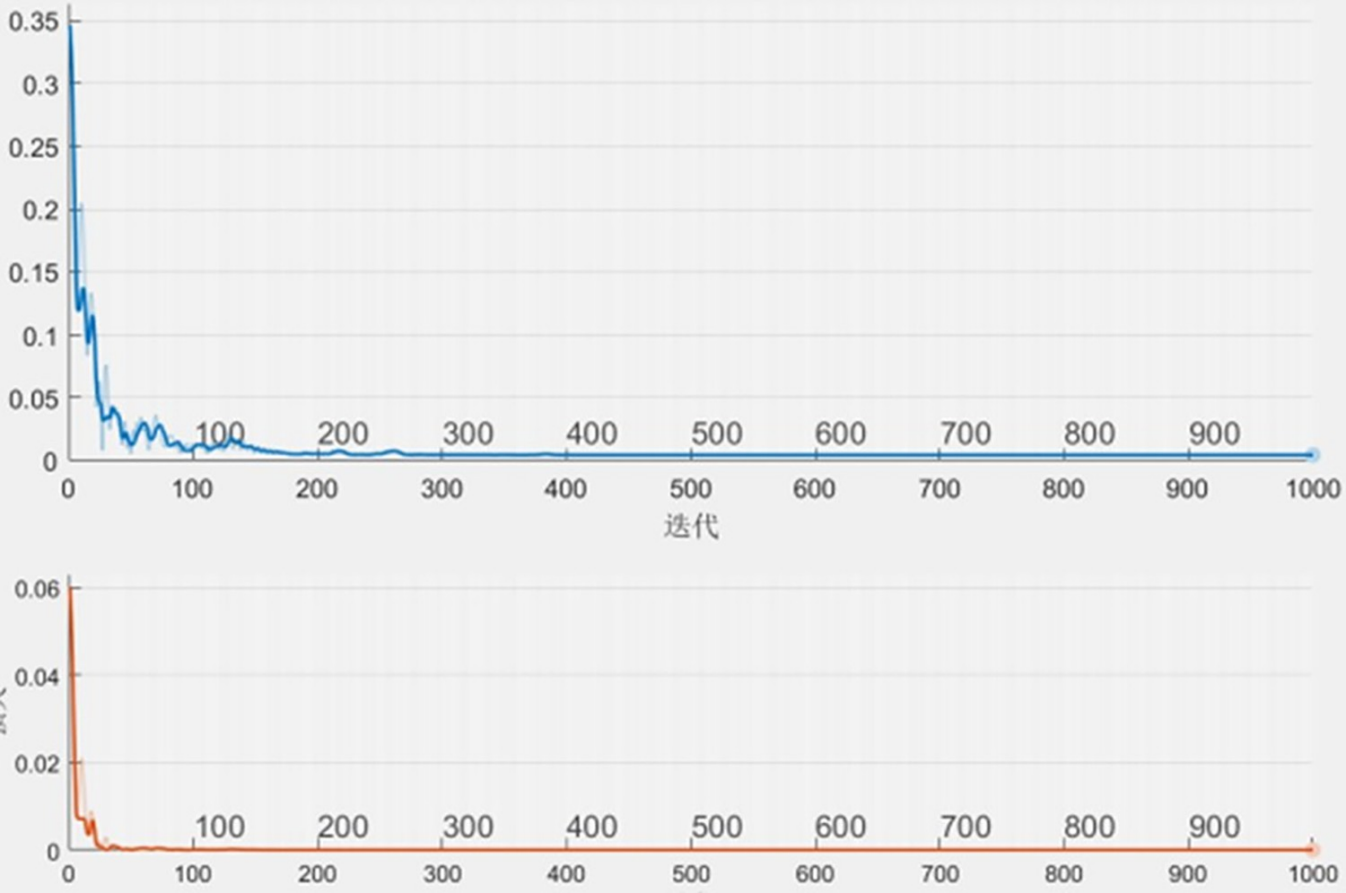
Wind speed modeling.

Run mainnew.m to get training and test results, including training and test loop network, generating training and test charts for each parameter, saving training and test results, actual results and error analysis in excel. Modeling with neural network is the modeling of four influence factors in turn. Take wind speed, for example. Wind speed modeling is shown in Fig 6.

The test results are stored in allDat.xlsx, and each column is respectively training or test data, actual data, relative error, the difference between training or test data and actual data after standardization, and root mean square error. Each parameter is placed on different pages.

When building a prediction model, the choice of model parameters directly determines the accuracy of the prediction results. Model parameters can be divided into two categories: one is the learning parameters such as the internal weight of the network, which are automatically learned and adjusted in the process of model training. The other is the parameters that need manual selection, such as the number of iterations, loss function, learning rate, etc., also known as hyper parameters.

At present, there is no complete set of methods to solve the selection of neural network super parameters, which are generally adjusted according to experimental results and parameter adjustment experience. In this paper, the size of root mean square error is used to judge whether the parameter selection is appropriate. From 2 to 4 neurons, 3 neurons in the input layer were finally determined. Through the test, each layer starts with 100 neurons, and the number of neurons in the hidden layer is determined to be 96*3. So three dimensions in, one dimension out. The solver is set to adam.

Gradient descent algorithm is adopted as the optimization algorithm. Gradient is a vector used to indicate the direction along which the value rises the fastest at a specific point of the function. This vector corresponds to the magnitude of the rising speed of the function value. The gradient threshold is set to 1. The gradient threshold is mainly used for gradient explosion. Even if the updated gradient exceeds this threshold, it will be restricted within this range.

The maximum number of training iterations is 1000. Due to the possibility of overfitting, the number of training should not be too large.

The amount of parameter updating during training is called the learning rate and is usually a positive value in the range of 0 to 1. The learning rate is used to control the updating speed of parameters when training neural networks. If the learning rate is low, the updating speed of parameters will be greatly reduced. When the learning rate is high, vibration will be generated in the search process, and the parameters will stay near the optimal value and not get good results. Through the test from 0.001, 0.005, 0.0001 finally determine the initial learning rate 0.005. The root mean square error is used as the loss function. The following takes the number of neurons in the input layer as an example to test the parameters. The root mean square error results of two neurons are as follows:

√(F1-y1)^2+(F2-y2)^2+(F3-y3)^2+(F4-y4)^2+…./n=√(16.5-15.24028)+(18-17.2305)+(19.21501-18)+…./38=3.684129

The root mean square error results of the three input neuron results are:

√(F1-y1)^2+(F2-y2)^2+(F3-y3)^2+(F4-y4)^2+…./n=√(16.5-15.24028)+(18-17.2305)+(19.21501-18)+…./38=3.506096

Each parameter selection neuron can see the root-mean-square error, according to the size of the root-mean-square error to judge the training and test effect, select better parameters. The final parameters are shown in Table 3.

**Table 3:** Final parameters.

Input layer neuron	Hidden layer neuron	Output layer neuron	Gradient threshold	maximum number of iterations	Initial learning rate
3	288	1	1	1000	0.005

After the super parameter is determined, the network model automatically adjusts and determines the weight parameters of neurons through the learning of the training sample set in the training process. The training process can be regarded as a process of constant adjustment of weight parameters. By learning the training sample set, the prediction model determines and saves the weight parameters inside the network. When the trained model inputs data with the same format as the sample data, it can predict and output the predicted value. Take latitude and longitude as an example. The average absolute error pairs between ordinary neural network and LSTM are shown in Table 4.

**Table 4:** Comparison of average absolute error between ordinary neural network and LSTM.

	General neural network predicts longitude of center position	General neural networks predict center location latitude	LSTM predicts the longitude of the center position	LSTM predicts the longitude of the center position
GAEMI	0.6922	0.3830	0.5924	0.3887
SARIKA	1.0474	0.7296	0.7395	0.4078
NIDA	0.6435	0.2863	0.5232	0.2640
KOMPASU	0.4236	0.3643	0.3087	0.2730
MERANTI	0.7211	0.5212	0.6345	0.3019
CONSON	0.9314	0.8824	0.7153	0.6821

It can be seen that the LSTM cyclic network model is more effective in predicting typhoon data based on the neural network model.

## 4. Case study

This paper analyzes the forecast data of Typhoon Chan-hom based on the model, which formed in the South China Sea on May 2, 2009. The impact of Chan-hom on China was mainly concentrated on the sea, where the wind and waves were high, which had a bad impact on fishing operations of fishing vessels and the navigation of merchant ships. The typhoon brought heavy rain to the northern Philippines, causing multiple house collapses and landslides, killing more than 25 people, leaving three others missing and hundreds of residents fleeing their homes. Ferry services on Luzon Island and several surrounding islands remained suspended as of Monday morning, leaving at least 1,000 passengers stranded at ferry crossings.

Typhoon intensity refers to the wind strength of the typhoon center. In China, typhoon intensity is classified according to the wind strength of the typhoon center, as shown in Table 5.

**Table 5:** Classification of tropical cyclones.

Tropical cyclone classification	Maximum mean wind speed (m/s)	Maximum wind force
tropical depression (TD)	10.8–17.1	6–7
tropical storm (TS)	17.2–24.4	8–9
severe tropical storm (STS)	24.5–32.6	10–11
typhoon (TY)	32.7–41.4	12–13
violent typhoon (STY)	41.5–50.9	14–15
super typhoon (SuperTY)	≥51.0	16 or above

The relationship between warning signals and wind strength is shown in Table 6.

**Table 6:** Relation between warning signal and wind strength.

Typhoon blue warning signal	Typhoon yellow warning signal	Typhoon orange warning signal	Typhoon red warning signal
Average wind strength is 6	Average wind strength is 8	Average wind strength is 10	Average wind strength is 12

**Fig 7:**
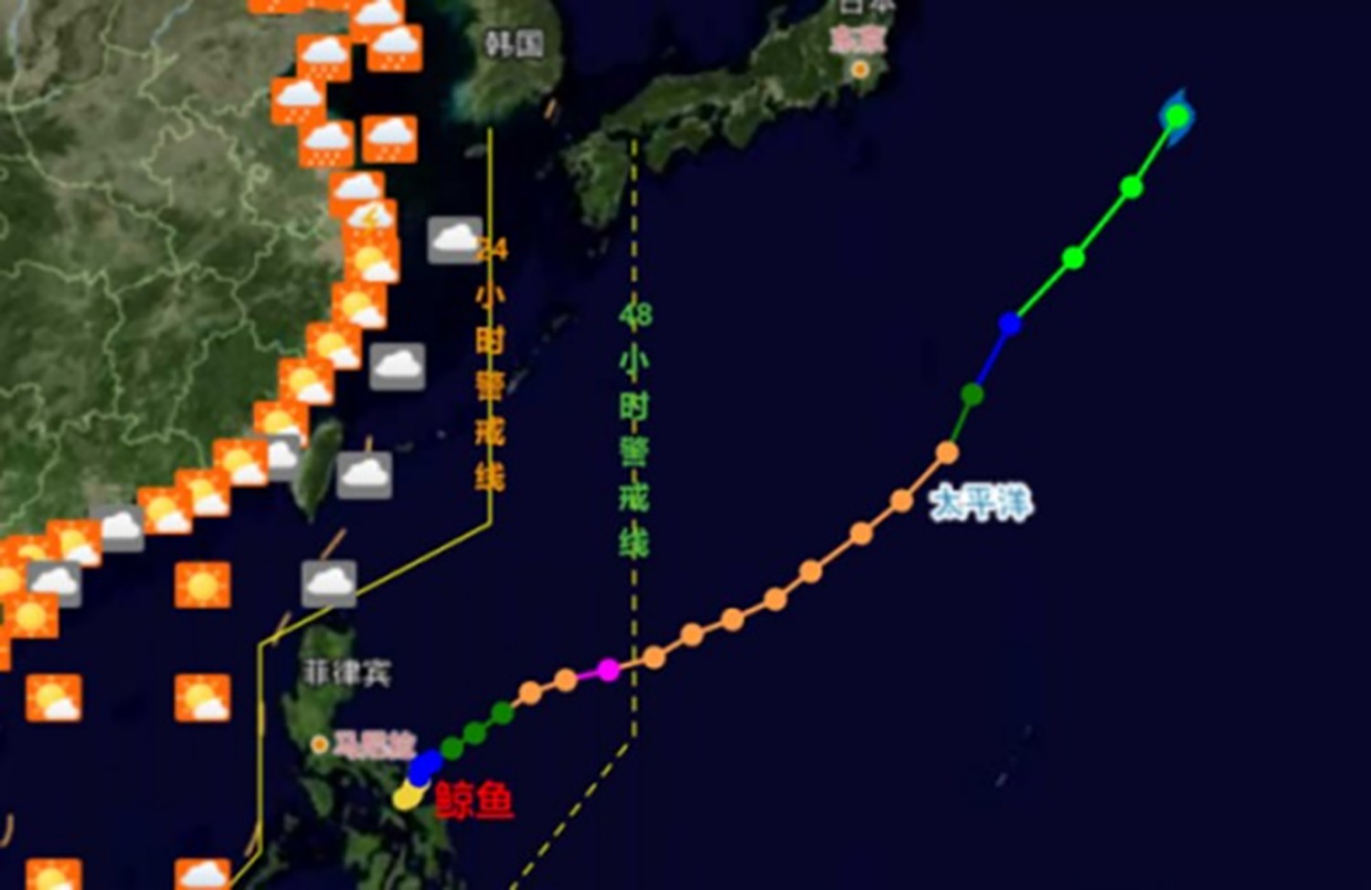
Warning signal colors on the web page.

The color of the warning signal is the same as that of each time point on the typhoon network, as shown in Fig 7.

The relationship between warning signals and emergency measures is shown in Table 7.

**Table 7:** Relationship of emergency measures for warning signals.

Typhoon blue warning signal	Typhoon yellow warning signal	Typhoon orange warning signal	Typhoon red warning signal
Be prepared for the wind. Stop dangerous outdoor operations at high altitudes	Go into windbreak and stop large gatherings	Emergency windproof state, stop large gatherings, shut down and suspend classes, transfer evacuation, personnel shelter	No assembly, no school, no shelter

As can be seen from the table, the blue typhoon warning has little impact on the public, the yellow typhoon warning has a low impact, and the orange typhoon warning and above has a great impact on the public.

### 4.1 Prediction and analysis of typhoon intensity data

The path data of typhoon Chan-hung was input, and the results were output through the model. The predicted value and the real value were analyzed by comparing the output to the typhoon intensity level corresponding to the maximum wind speed and central pressure. Some data such as maximum wind speed and central pressure are shown in Table 8.

**Table 8:** Maximum wind speed, central pressure and other partial data.

Predicted maximum wind speed	True maximum wind speed	Predicted central pressure	True central pressure	The predicted value corresponds to the wind strength rating		The true value corresponds to the wind level	The predicted value corresponds to the typhoon intensity level	The true value corresponds to the typhoon intensity level
16.24212456	18	995.5469971	997	6–7		8–9	tropical depression (TD)	tropical storm (TS)
18.70079231	18	996.383728	996	8–9		8–9	tropical storm (TS)	tropical storm (TS)
20.45780945	19	995.2406616	995.5	8–9		8–9	tropical storm (TS)	tropical storm (TS)
. . .	. . .	. . .	. . .	. . .		. . .	. . .	. . .
29.31224632	33	980.7211304	975	10–11		12–13	severe tropical storm (STS)	typhoon (TY)
29.74018097	34	979.2316284	972.5	10–11		12–13	severe tropical storm (STS)	typhoon (TY)
29.99897003	32.5	978.1166992	972.5		10–11	10–11	severe tropical storm (STS)	强热带风暴(STS)
29.46956635	27.5	978.0719604	982.5		10–11	10–11	severe tropical storm (STS)	强热带风暴(STS)
27.33906555	25	982.2059326	990		10–11	10–11	severe tropical storm (STS)	强热带风暴(STS)
24.71959114	24	988.4030762	990		10–11	8–9	severe tropical storm (STS)	tropical storm (TS)

According to the true value of the chart, the relationship between the maximum wind speed and the central pressure is as follows: the greater the central pressure is, the lower the wind speed is. According to the predicted value, the best fitting effect is achieved by strong tropical storm, and the bad forecasting effect of tropical depression, typhoon and strong typhoon should be improved. The lower the central pressure of the typhoon, the greater the pressure gradient, pressure difference and height difference, the greater the corresponding wind, and the higher the typhoon intensity. The rapid drop in pressure indicates that typhoon intensifies and develops rapidly. For example, after super Typhoon Meranti made landfall on 14th, 2016, the pressure in Kinmen County, Fujian Province dropped by 8.8hpa within 15 minutes. If the central pressure is lower than 975hpa and the corresponding wind speed is 33m/s, it meets the standard of Category 12 typhoon. Below 900kpa corresponds to wind speeds of 70m/s, which is considered a Category 5 hurricane. The strongest typhoon ever observed was Tip, located over the northwest Pacific Ocean at 23:00 on October 12, with a central pressure of 870kpa corresponding to a wind speed of 85m/s. This record has not been broken to this day.

The calculated average absolute error of wind speed prediction is:

(F1-y1)+(F2-y2)+(F3-y3)+(F4-y4)+… ./n = (18–16.24212456)+(18.70079231–18)+(19–20.45780945)+… ./38 = 2.39077, this indicates that the average deviation between the predicted wind speed and the actual wind speed is about 2.4m/s, and the root mean square error is 3.506096.

The average absolute error of central pressure forecast by calculation is:

(F1-y1)+(F2-y2)+(F3-y3)+(F4-y4)+… ./n = (997–995.5469971)+(996.383728–996)+(995.5–995.2406616)+… ./38 = 2.48236, this indicates that the average deviation between the predicted typhoon central pressure and the real typhoon central pressure is about 2.48 hpa, and the root mean square error is 3.622991.

**Fig 8:**
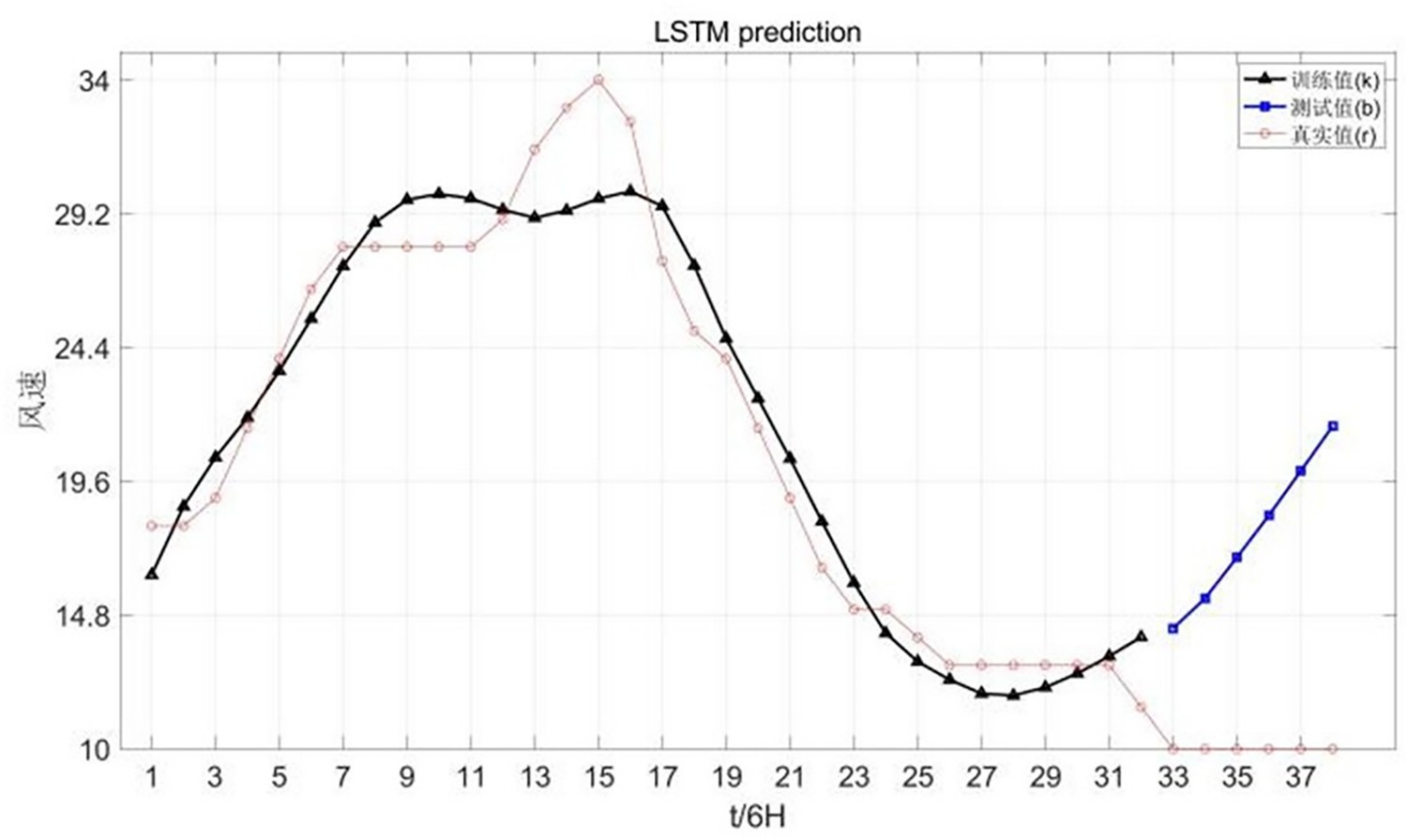
Canchan-Hom wind speed forecast chart.

Chan-hom wind speed forecast is shown in Fig 8.

**Fig 9:**
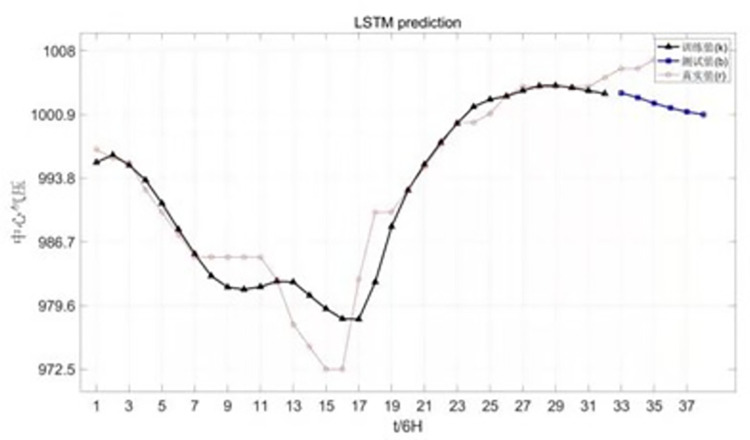
Central pressure forecast of Chan-hom.

The forecast chart of Chan-hom central pressure is shown in Fig 9.

### 4.2 Typhoon location data prediction and analysis

Table 9 shows the predicted and true data of latitude and longitude of the central location predicted by the model.

**Table 9:** Predicted data and true data statistics of latitude and longitude of the central location.

Predicted latitude of center location	True latitude of center position	Predicted longitude of center position	True longitude of the center position
10.34400558	10.4	112.7157669	112.4
10.7035408	10.7	112.2525406	112.6
11.06176281	11	112.1345825	112.7
11.42400742	11.4	112.2401733	112.6
11.78918076	11.6	112.437027	112.6
12.146842	11.8	112.6790237	112.6
12.48476505	12.1	112.9611435	112.5
. . .	. . .	. . .	. . .
17.30318832	17.4	123.5119324	124
17.73790932	17.4	124.8717575	125.3
18.107687	17.3	126.112381	126.4

The calculated average absolute error of longitude prediction of the central position is:

(F1-y1)+(F2-y2)+(F3-y3)+(F4-y4)+… ./n = (112.7157669–112.4)+(112.6–112.2525406)+(11.06176281–11)+… ./38 = 1.12374, this indicates that the average deviation between the predicted longitude of the typhoon center and the true longitude of the typhoon center is about 1.12 degrees, and the root mean square error is 2.226755.

The calculated average absolute error of latitude prediction of the central location is:

(F1-y1)+(F2-y2)+(F3-y3)+(F4-y4)+… ./n = (10.4–10.34400558)+(10.7035408–10.7)+(19–20.45780945)+… ./38 = 0.51073, this indicates that the deviation between the predicted latitude of the typhoon central location and the true latitude of the typhoon central location is about 0.51 degrees on average, and the root mean square error is 0.714784.

**Fig 10:**
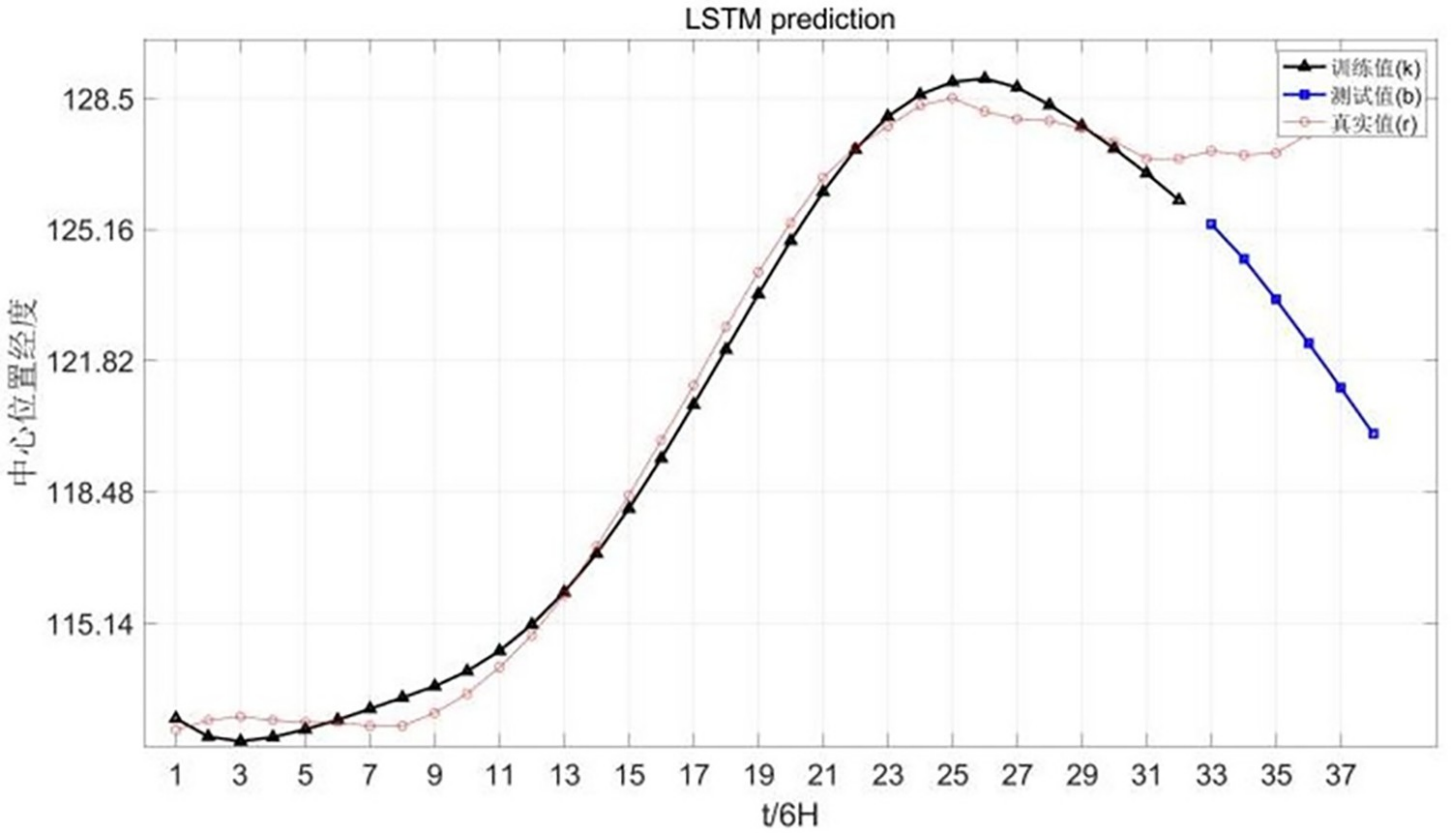
Longitude prediction of Chan-hom central position.

The longitude prediction diagram of Chan-hom central position is shown in Fig 10.

**Fig 11:**
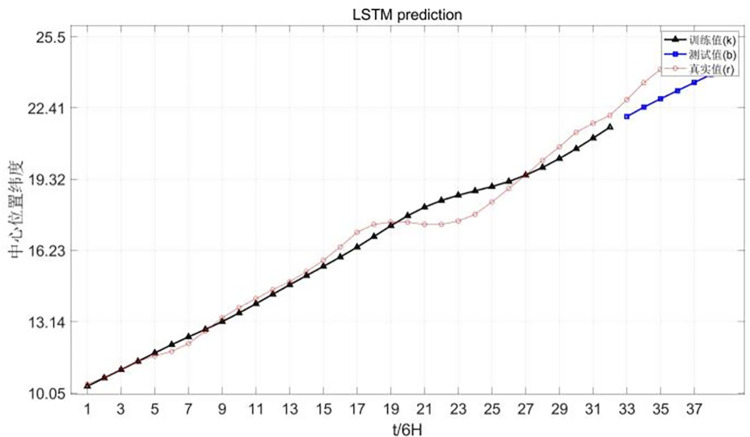
Latitude prediction of Chan-hom’s central location.

The latitude prediction chart of Chan-hom central location is shown in Fig 11.

The results show that the latitude fitting is the best in the center of the model and the wind speed is the lowest.

**Fig 12:**
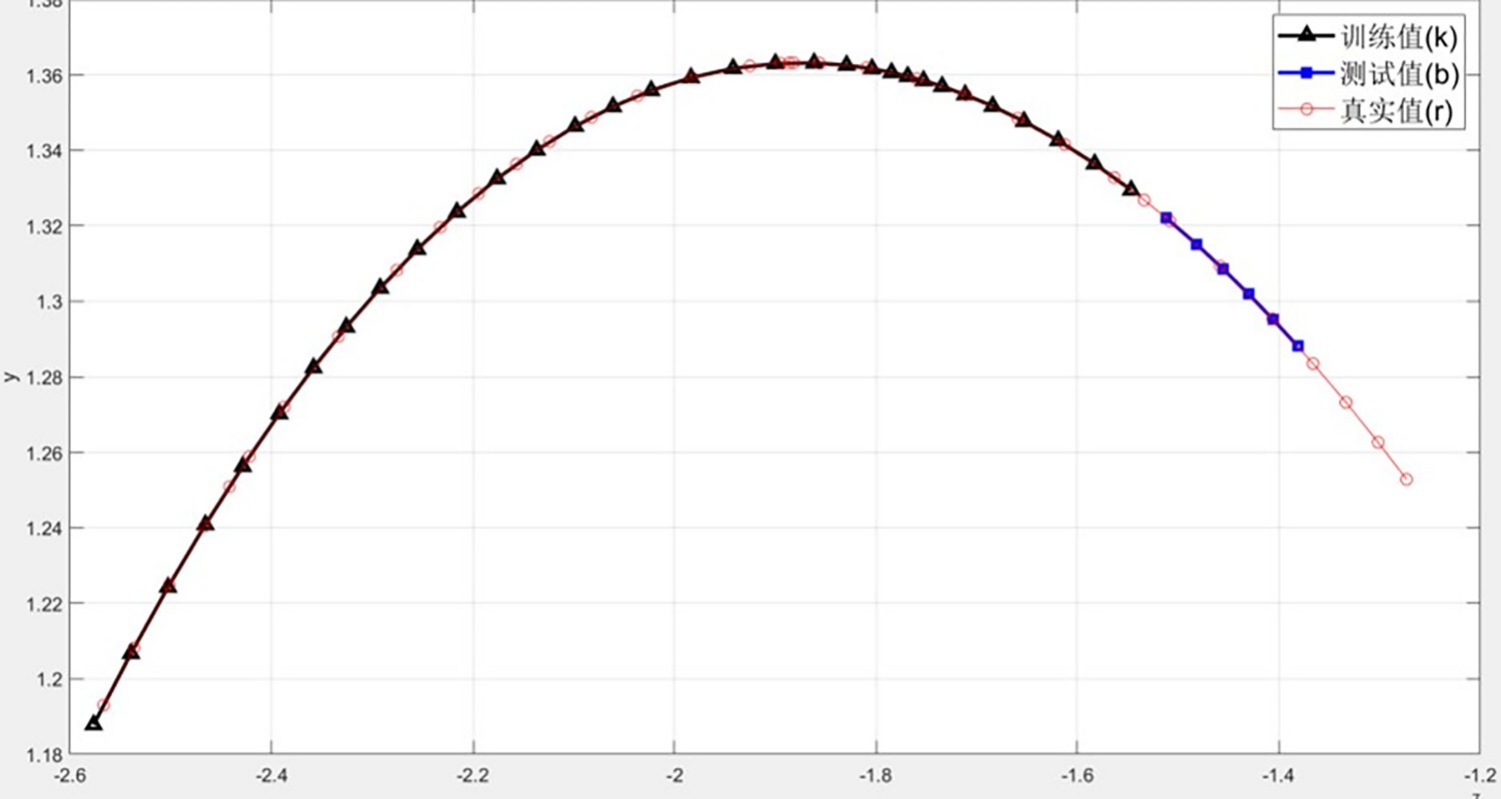
Typhoon track chart.

The longitude and latitude of the central position longitude and latitude data in alldata1.xlsx were converted to x y [X, Y], and the track chart of Typhoon Chan-hom was obtained, as shown in Fig 12.

According to the path diagram, the predicted value is basically consistent with the real value.

### 4.3 Analysis of disaster trend data

Typhoon disaster trend statistics in Western Pacific.

**Fig 13:**
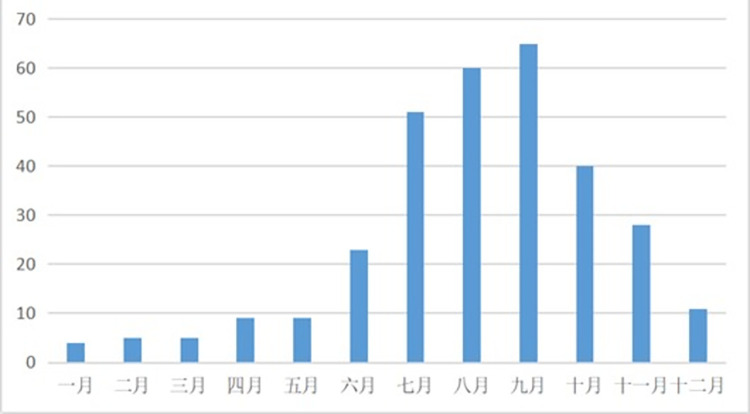
Change in the number of typhoons per month from 2009 to 2019.

The Western Pacific Ocean adjacent to China is a region with a high incidence of typhoons in the world, so it is of great significance to study its characteristics for China’s typhoon disaster emergency prediction. According to the typhoon track data set, the analysis results are as follows:

There is a big difference in the number of typhoons occurring every month. The average number of typhoons occurring every month is about 26. The number of typhoons occurring in September is the highest, reaching 65, while the number of typhoons occurring in January is the lowest, only 4. The change of the number of typhoons occurring every month from 2009 to 2019 is shown in Fig 13.

**Fig 14:**
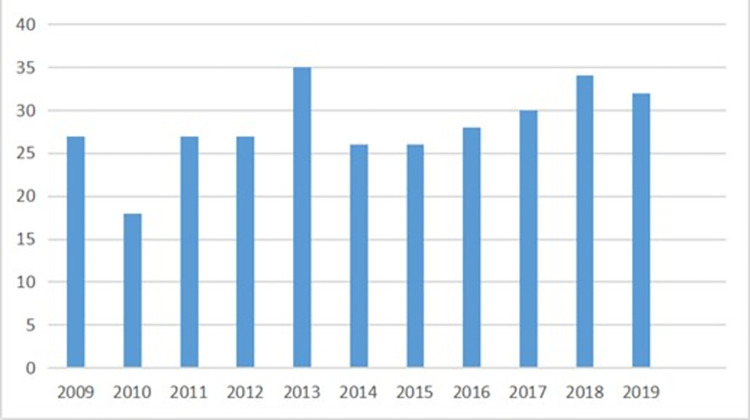
Annual change in the number of typhoons from 2009 to 2019.

The average number of typhoons every year is about 28. In 2013, the maximum number of typhoons is 35. In 2010, the minimum number of typhoons is 18. The annual change in the number of typhoons from 2009 to 2019 is shown in Fig 14.

Typhoon disaster trend statistics in China.

According to the typhoon landing data set, the characteristics of typhoons landing in Chinese provinces and cities are analyzed from three aspects: location, intensity and time. No typhoon landing records in Hebei Province are not included in the charts and tables.

According to the statistics of typhoon landing intensity (if the same typhoon landing in multiple places is recorded as the one with high intensity), the statistics of typhoon landing intensity of coastal provinces and cities in China from 1945 to 2015 are shown in Table 10.

**Table 10:** Typhoon landing intensity statistics of coastal provinces and cities in China from 1945 to 2015.

	Liao ning	Tian jin	Shan dong	Jiangsu	Shanghai	Zhe jiang	Fu jiang	Guang dong	Guangxi	Hainan	Taiwan
tropical depression (TD)	4	1	4	2	2	2	7	20	1	16	5
tropical storm (TS)	3	0	5	0	5	5	23	39	7	17	8
severe tropical storm (STS)	4	0	7	1	4	11	37	75	10	29	25
typhoon (TY)	0	0	0	2	2	15	34	55	2	37	44
violent typhoon (STY)	0	0	0	0	0	9	6	17	1	9	36
super typhoon (SuperTY)	0	0	0	0	0	2	0	2	0	2	14
Total	11	1	16	5	13	44	107	208	21	110	132

As can be seen from the above table, Liaoning Province, Tianjin City and Shandong Province have no typhoon, strong typhoon or super typhoon landing records; Jiangsu Province and Shanghai have no strong typhoon or super typhoon landing records; Fujian Province and Guangxi Province have no super typhoon landing records; other provinces and cities have typhoons of various intensities. The number of landfalls recorded was tied for the most severe tropical storm category and tropical depression category. In terms of the total number of typhoons, Guangdong Province has the largest total number of typhoons, accounting for 31.1% of the total number of typhoons in China, while Taiwan Province, Hainan Province and Fujian Province account for 19.8%, 16.5% and 16% respectively. In typhoon season, high vigilance is also needed. Although there may not be one typhoon landing in Zhejiang Province, Guangxi Province, Shanghai Province and Jiangsu Province every year on average, However, due to the occurrence of typhoon level and above, so also need to pay attention to.

Table 11 shows the seasonal statistics of typhoon landings of coastal provinces and cities from 1945 to 2015.

**Table 11:** Seasonal statistics of typhoon landfall in China’s coastal provinces and cities from 1945 to 2015.

	Liao ning	Tian jin	Shandong	Jiangsu	Shanghai	Zhe jiang	Fu jian	Guangdong	Guangxi	Hainan	Tai wan
First	0	0	0	0	0	0	0	0	0	0	0
Second	0	0	1	0	1	2	5	34	11	15	15
Third	11	1	17	5	12	40	95	150	10	71	108
Forth	0	0	0	0	0	2	7	24	0	24	9

According to the table above, the typhoon season in Liaoning Province is the third quarter. Only one typhoon was recorded in Tianjin, which occurred in July, the third quarter. The typhoon season in Shandong Province is in the second and third quarters, with the most concentrated in July and August, accounting for about 94%. The typhoon season in Jiangsu Province is the third quarter, with the most concentrated in August, accounting for about 80%. The typhoon season in Shanghai is the second and third quarters, in which the third quarter is the most concentrated, accounting for about 92%. The typhoon season in Zhejiang Province was in the second, third and fourth quarters, with the most concentrated in July and August, accounting for about 72%. The typhoon season in Fujian Province was in the second, third and fourth quarters, among which the third quarter was the most concentrated, accounting for about 89%. The typhoon season in Guangdong Province covers the second, third and fourth quarters, among which the third quarter is the most concentrated, accounting for about 73%. The typhoon season in Guangxi Province is in the second and third quarters, of which the third quarter is the most concentrated, accounting for about 71%. The typhoon season in Hainan Province covers the second, third and fourth quarters, with the most concentrated period from July to October, accounting for about 79%. The typhoon season in Taiwan Province covers the second, third and fourth quarters, among which the third quarter is the most concentrated, accounting for about 82%. In general, all provinces and cities had no typhoon record in the first quarter, and typhoons were most likely to occur in the third quarter, especially in July and August.

## 5. Conclusions

This study develops a typhoon disaster emergency prediction method based on big data. Firstly, through numerical analysis of existing typhoons and analysis of typhoon disaster trends, this paper attempts to explore the laws of typhoon disasters. Secondly, in the analysis process, this study uses ordinary neural networks and LSTM models to predict the likelihood of typhoon disasters occurring in a certain period in the future. After numerical analysis, according to the prediction results of the LSTM model, the latitude fitting at the center of the model is the best, and the wind speed is the lowest. The average error of longitude and latitude at the center position is relatively small, and the predicted value of the path map is basically consistent with the actual value.

Based on the proposed model, the practical significance of this study is summarized as follows. Firstly, the average number of typhoons in the Western Pacific is approximately 26 per month and 28 per year. Secondly, this study predicts that typhoons landing in China are most likely to occur in the third quarter, with Guangdong Province accounting for the largest number of typhoons, accounting for 31.1% of the total number of typhoons in the country. Other coastal provinces and cities also need to pay attention to the typhoon season. On average, 9 typhoons land in China every year, of which 43.3% are typhoons above typhoon level.

To improve the theoretical system of typhoon emergency prediction, natural disaster is one of the practical problems restricting social development, is the object of all kinds of statistical prediction, typhoon disaster is one of the important forms of expression, but also one of the most serious natural disasters affecting our country, with strong seasonality, strong destructive power, wide coverage, prevention difficulties and other characteristics. Every year, typhoons bring abundant rainfall to the land, but also cause huge loss of people and property, so it is of great significance to analyze and predict the degree of typhoon disaster in time. Strengthen the public’s cognition of typhoon disaster theory, promote the public to understand typhoon disasters, identify disaster risks, enhance the awareness of typhoon prevention, and then spontaneously improve the ability to avoid and save themselves, so that the public is more likely to protect their personal safety and property safety when the typhoon comes. To provide assistance to disaster prevention and reduction departments, research on typhoon disaster emergency prediction methods based on big data will provide basis and support for statistical prediction of typhoon disasters, improve the speed of typhoon disaster emergency response, make relevant disaster prevention and reduction departments better prepared, and reduce personnel and property losses.

Finally, due to the lack of consideration of secondary and derivative disasters caused by typhoons, the proposed model of this study has certain limitations. First of all, this paper only forecasts one type of typhoon, and does not compare the forecast results of different types of typhoons, which will be improved in the future. Secondly, according to the analysis of forecast results, the fitting effect of strong tropical storm is the best, and the bad forecasting effect of tropical depression, typhoon and strong typhoon should be improved. Finally, typhoons may lead to other disasters such as heavy rain and debris flow, and the research on the correlation between other typhoons and other disasters is not involved in this paper.
